# Augmented reality (AR) in microsurgical multimodal image guided focal pediatric epilepsy surgery: Results of a retrospective feasibility study

**DOI:** 10.1016/j.bas.2024.104180

**Published:** 2025-01-11

**Authors:** Julia Shawarba, Matthias Tomschik, Jonathan Wais, Fabian Winter, Christian Dorfer, Florian Mayer, Martha Feucht, Karl Roessler

**Affiliations:** a Neurosurgical Clinic, Medical University Vienna, Austria; b Clinic for Pediatrics and Adult Medicine, Medical University Vienna, Austria

**Keywords:** Augmented reality, Image guided surgery, Pediatric epilepsy surgery, Seizure outcome

## Abstract

**Introduction and research question:**

Augmented reality (AR) is increasingly being used to improve surgical planning and assist in real time surgical procedures. A retrospective investigation was conducted to study its feasibility in pediatric epilepsy surgery at a single institution.

**Methods:**

Functional neuronavigation using multimodal imaging data (fMRI, DTI-tractography, PET, SPECT, sEEG) were used to augment the surgical navigation by transferring MRI imaging reconstructions as AR maps into the surgical microscope overlaying the surgical field.

**Results:**

Altogether, 43 patients (17 female, 0–18 yrs, mean 9 yrs) were operated between 10/2020 and 10/2023 and fulfilled the inclusion criteria. 26 patients (60.5%) had an extra-temporal and 17 (39.5%) a temporal seizure origin. The 3 top histological diagnoses encountered were: FCD (32.6%), ganglioglioma (23.3%) and DNET (11.6%). Preoperative MRI studies showed no epileptogenic lesion in 11 patients (25.6%, MRI negativ group), which necessitated implantation of depth electrodes before resection. There were no adverse events while using AR enhanced neuronavigation. Altogether, of 24 patients with a follow up of more than one year, 83.3% displayed a favorable ILAE grade 1 seizure outcome (75% ILAE 1a), 14 % experienced a transient hemiparesis, 4.3% a permanent quadrantanopia and one needed a subdural-peritoneal shunt.

**Discussion and conclusion:**

AR supported navigated microscope resection allowed targeting and removal of lesional as well as non-lesional (sEEG defined) epileptogenic zones in pediatric epilepsy surgery with low morbidity and an expected seizure outcome.

## Highlights

• Effects of use of augmented reality in pediatric epilepsy surgery was investigated.
• Multimodal imaging was inserted in the head-up display of the microscope.
• 83% of 43 patients had a favorable ILAE grade 1 seizure outcome (75% ILAE 1a).
• AR supported resection facilitated targeting and removal of epileptic lesions.

## 1 Introduction

In pediatric epilepsy surgery the definition of the epileptogenic zone (EZ), the brain tissue which has to be removed to render the patient seizure free, is still a challenge and needs multiple preoperative image investigations for definition of the surgical resectability even in focal epilepsy (Gong et al., 2024). Currently, state of the art neuronavigation serves as a tool for transferring image information onto the operative field using pointer devices or border contours displayed within the surgical microscope (Roessler et al., 1998a, 1998b). Unfortunately, this tool strategy does not support a simultaneous visual assessment of the radiological information of the image screen of the navigation system and the surgical manipulation on the patient's tumor-/brain tissue within the operative field (Roessler et al., 1998b). Augmented reality overlays digital information on to the surgeon's real-world view by combining imaging data like structural and functional MRI scans with live views of the surgical field by using advanced microscope systems (Kos et al., 2023). Augmented reality (AR) is also increasingly used for surgical education, but already also for guiding intraoperative surgical strategy in different fields of neurosurgery, like spine surgery, skull base surgery and vascular neurosurgery (Begagic et al., 2024; Fabrig et al., 2024; Halpin et al., 2024; Judy et al., 2024; Kos et al., 2023; Nguyen et al., 2024).

**Fig. 1:**
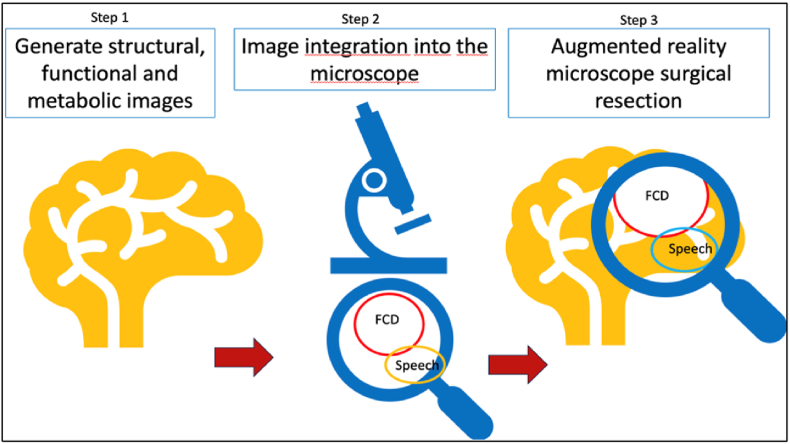
Step1: imaging, Step 2: image integration into the microscope, Step 3: AR resection.

**Fig. 2:**
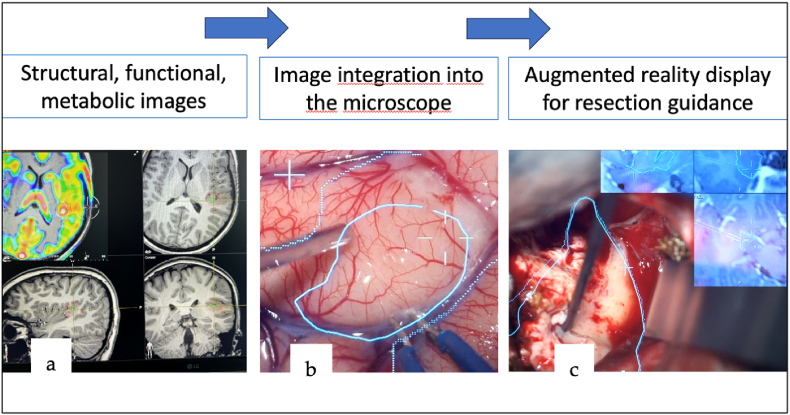
Patient 10 with the left temporo-parietal DNET: Hypermetabolic area on the FDG PET (a), transferred to the microscope as a contour and projected on to the cortex (b), intraoperative guidance using the 3-plan-MRI AG during resection (c).

Putative epileptogenic zones created out of stereo-electro-encephalography (sEEG) results, images from positron emission tomography (PET) and single-photon emission computer tomography (SPECT) may be directly used for image guided surgery (IGS). Therefore, images have to be overlayed on the surgical field for guiding the resection, typically using the microscopes head up display (HUD) (Roessler et al., 2024) (Fig. 1). Additionally, augmented reality support can be performed by using surgical instruments like a navigated suction device additionally to the multicolor display of the surgical microscope to transfer image information on to the brain of the surgical field during resection, respectively. MRI scans including multimodal imaging information are used to augment the resection strategy further by displaying it additionally in the right upper corner of the eyepiece (HUD) of the microscope (Fig. 2).

However, the effectivity and results of such an augmented reality supported resection strategy in pediatric epilepsy surgery has not yet been investigated or shown to be beneficial till now. Therefore, a retrospective study was performed to analyze the direct impact on the resections of lesions/epileptogenic zones as well as on complications and the resulting seizure outcome.

The study was conducted in accordance with the Declaration of Helsinki, and approved by the Institutional Review Board (or Ethics Committee) of Medical University of Vienna (EK Nr: 1542/2024, ARPES Study-study on the effect of “augmented reality in pediatric epilepsy surgery”).

## 2 Materials and methods

### 2.1 Augmented reality application, neuronavigation, intraoperative MRI

The neurosurgical department, MUW (Medical University Vienna) has performed pioneer work in application of neuronavigation in neurosurgery in the past: mechanical and infrared pointer systems and a robotic microscope system (MKM Zeiss, Oberkochen, Germany) were introduced in 1994, investigating its applicability for neurosurgical procedures (Roessler et al., 1998a, 1998b). The new microscope generation was introduced 2019 (Zeiss Kinevo Microscope®, Oberkochen, Germany), which allows the implementation of augmented reality via a multicolor microscopical display (Head up display-HUD) using a commercial software.

Moreover, we integrated an intraoperative high field (3T) MRI (Magnetom Skyra, Siemens Healthineers, Germany) as a two-room set-up at our institution in October 2020 (Roessler et al., 2024). As a navigation system, the Brainlab® Navigation System (Brainlab, Munich, Germany) was connected to the Zeiss microscope and used as a neuronavigation tool. In addition, for providing resection contours, multiplanar and 3 plane MRI images can be displayed in the microscopical eye piece and used as a guide for navigated resection (Fig. 2). This allows the simultaneous view of overlayed augmented reality images in the HUD on to the operating field and the simultaneous surgical manipulation on the tissue and the assessment of the imaging view of the manipulated tissue by being able constantly looking through the microscope (Fig. 1).

Moreover, the Brainlab® Navigation allows to implement additional instruments as navigation tools for the resection, like a navigated suction device. For that study, a regular suction device (Neuromedics, Hamburg, Germany) was equipped with Brainlab® tracking stars and implemented in the navigation as a surgical navigation tool. The tip of the suction could also be displayed on the virtual reality images overlayed in the eyepiece of the microscope and used for navigational guidance during resection (Augmented reality instrument navigation support).

### 2.2 Pediatric epilepsy surgery patients

Pediatric epilepsy surgery is an important focus of the Neurosurgical Department, Medical University of Vienna, including approximately 40 resection/disconnection surgeries, 8–10 depths electrode implantations and 10 VNS implantations per year on average. The retrospective patient population investigated was chosen according to the following inclusion criteria: 1. patient age from 0 to 18 years, 2. medically refractory epilepsy, 3. definition of a focal area as a putative epileptogenic zone by video- EEG monitoring and by preoperative image investigations (MRI, PET, SPECT) for resection, or 4. invasive long-term monitoring by using depths electrodes in MRI negative cases (sEEG).

Patients were investigated preoperatively in the pediatric epilepsy monitoring unit using neurological investigation as well as clinical and video EEG monitoring as described earlier (Shawarba et al., 2024). Resection was performed using standard microsurgical techniques supplemented by neuronavigation including augmented reality and intraoperative MRI and intraoperative electrocorticography (ECoG)as well as functional MRI for motor and speech paradigm and DTI for internal capsule and arcuate fascicle, in detail described before (Sommer et al., 2013, 2014, 2015), Fig. 1.

### 2.3 Intraoperative sEEG, ECoG and iopMRI

Seven patients (16.3%) had preoperative sEEG by depth electrodes, due to MRI negative putative EZs (6 patients) or because of lesions unclear of causing the focal epilepsy (1 patient). In these patients, the putative EZ which was planed to resect was manually segmented according to the positive contacts of the depth electrodes preoperatively, Table 1. In 34 of 43 patients (79%, Table 1), intraoperative electrocorticography (ECoG) was used for pre- and post-resection EEG assessment and, when feasible, for tailoring the amount of resection, according to ILAE recommentations (Greiner et al., 2016; Jayakar et al., 2016). In 38 of the 43 patients (88.4%), an intraoperative MRI was performed, which resulted in a second look surgery in 3 patients (7.9%), where residual lesions were resected after iopMRI.

**Table 1:** Patients demographics and results.

Pat.Nb.	Age	MR lesion localization	MRI neg	sEEG	Surgical intervention	Histology	Resektion amount	ECoG	ioMRT	Reoperation	Epilepsy Outcome	Complications
1	16	left frontal			FLR	AngiozGliom	total	1		1	Wieser 1a	
2	15	left temporal			tailored Lesionectomy	DNT	total	1	1		Wieser 1a	
3	14	left temporal			AMTLR	mMCD2	total		1		<1 yr FU	
4	10	right frontal		1	Tailored lesionectomy	FCD2a	total	1	1		Wieser 1a	
5	12	left frontal	1	1	Tailored lesionectomy	FCD2a	total	1	1		<1 yr FU	intermitt HP
6	11	left frontal			Tailored FLR	FCD	sub	1			Wieser 1a	
7	9	left temporal			tailored lateral TLR	GG	sub	1	1		Wieser 1a	
8	6	left temporal			Tailored TLR	GG	total	1	1		<1 yr FU	
9	12	left frontal			FLR	Gliose	sub	1	1		<1 yr FU	intermitt HP
10	17	right parietal			Tailored resection	DNT	total	1			Wieser 1a	
11	3	left frontal	1	1	FLR	FCD2b	total	1	1		Wieser 1a	
12	0	right temporal			Tailored lesionectomy	SEGA/FCD2b	total	1	1		Wieser 1	VP Shunt
13	4	right temporal			TLR	HS	total	1			<1 yr FU	
14	4	right temporal			AMTLR	GG	total		1		Wieser 1a	
15	9	right insular			Tailored lesionectomy	Astroblastom	total	1	1		<1 yr FU	
16	14	left frontal			Lesionectomy	DNT	total		1		Wieser 1a	
17	12	right insular	1	1	Tailored lesionectomy	FCD	sub	1	1		Wieser 4	
18	0	right parietal			Tailored lesionectomy	FCD2b	total	1	1		<1 yr FU	intermitt HP
19	17	right frontal	1	1	Tailored AMTLR	mMCD2	total		1		Wieser 4	intermitt HP
20	11	left temporal			AMTLR	GG	total	1	1		<1 yr FU	Quadrantanopia
21	0	left temporal			Tailored lesionectomy	FCD2b	total	1	1		<1 yr FU	
22	7	left insular	1		tailored lesionectomy	Enc	total	1	1		<1 yr FU	
23	16	left frontal	1	1	tailored lesionectomy	FCD2a	total	1			Wieser 1a	
24	4	left frontal			AMTLR	GG	total		1		Wieser 1a	
25	10	left frontal	1		FLR	MOGHE	total		1		<1 yr FU	
26	9	left temporal			tailored lesionectomy	Gliose	sub	1	1		Wieser 1a	Quadrantanopia
27	18	right temporal			AMTLR	GG	total	1	1	1	<1 yr FU	
28	13	left temporal			AMTLR	GG	total	1	1		<1 yr FU	
29	4	right frontal			FLR	MOGHE	total	1	1		<1 yr FU	
30	0	left parietal			lesionectomy	DIG/DIA	total				<1 yr FU	
31	11	right parietal			Tailored lesionectomy	DNT	total	1	1		<1 yr FU	
32	0	right insular			tailored lesionectomy	FCD2b	total	1	1		Wieser 1a	
33	14	right temporal	1		Tailored lesionectomy	FCD2b	sub	1	1		Wieser 4	
34	14	left frontal	1		Tailored lesionectomy	FCD2a	total	1	1		Wieser 1a	intermitt HP
35	3	left frontal	1	1	Tailored lesionectomy	FCD2b	sub	1	1		Wieser 1a	intermitt HP
36	15	right temporal	1		AMTLR re	mMCD2	total	1	1		Wieser 2	
37	6	left frontal			Tailored lesionectomy	Cavernom	total	1	1		Wieser 1a	
38	3	left temporal			AMTLR	GG	total		1	1	Wieser 1a	
39	5	right frontal			FLR	FCD2b	total	1	1		<1 yr FU	
40	18	left frontal			Tailored lesionectomy	DNT	total	1	1	1	Wieser 1	
41	16	right occipital			Tailored lesionectomy	GG	total		1		<1 yr FU	
42	2	right temporal			Tailored lesionectomy	GG	total	1	1		Wieser 1a	CSF accumulation
43	4	right parietal			Tailored lesionectomy	FCD2a	total	1	1		<1 yr FU	

Pat.Nb.: patient number, FLR: frontal lobe resection, AMTLR: antero-mesial tempral lobe resection, TLR: temporal lobe resection, intermitt HP: intermittend hemiparesis.

### 2.4 Patients demographics

Altogether, 43 patients (17 female, 0–18 yrs, mean 9 yrs) were operated between 10/2020 and 10/2023 and fulfilled the inclusion criteria. 26 patients (60.5%) had an extra-temporal and 17 (39.5%) a temporal seizure origin. The exact localization of the lesions was: 17 temporal, 17 frontal, 5 parietal, 3 insular, 1 occipital. The histological diagnoses of the resected tissue were: 17 FCD2ab (focal cortical dysplasia)/mMCD (mild malformation of cortical development), 15 GG/DNET (ganglioglioma, dysembrioplastic neuroepithelioma), 2 MOGE (mild malformation of cortical development with oligodendroglial hyperplasia), 2 Gliosis, 1 angiocentric glioma, 1 desmoplastic infantile ganglioglioma, 1 immune-encephalitis, 1 cavernous malformation, 1 subependymal giant astrocytoma, and 3 others, Table 1. Therefore, the 3 top histological diagnoses encountered were: FCD (32.6%), ganglioglioma (23.3%) and DNET (11.6%).

Preoperative MRI studies showed no epileptogenic lesions in 11 patients (25.6%, MRI negative group), which necessitated implantation of depths electrodes before resection in 6 of these patients, the other 5 were resected according to the PET hypometabolism (Table 1). The histological diagnoses of the MRI negative cases were: FCD in 7 cases, mMCD in 2, encephalitis in 1, MOGHE in 1.

### 2.5 Augmented reality planning, neuronavigation and intraoperative MRI workflow

**Fig. 3:**
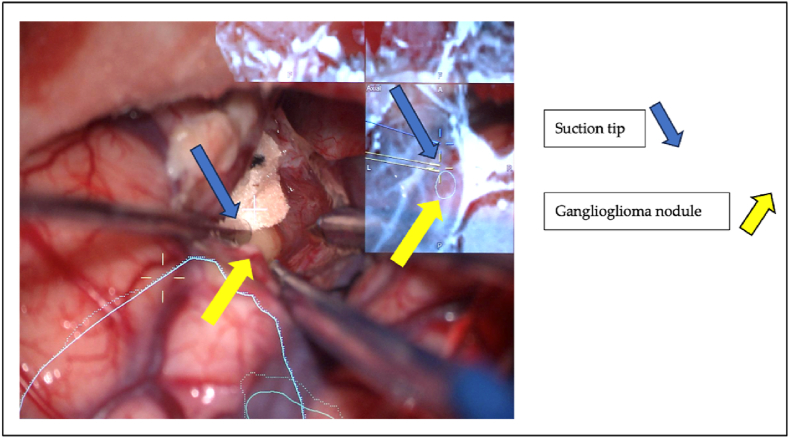
Tailored resection of a right temporo-mesial ganglioglioma: the suction tip is navigated and seen on the 3-plan AR MRI images as a yellow pointer with a yellow cross on the tip. This correlated well with the yellow ganglioglioma seen on the intraoperative fotograph.

The structural, metabolic and functional imaging was segmented within the navigation system (Brainlab, Munich, Germany) and displayed as contours on the MRI images, which consequently were transmitted into the eyepiece of the microscope (HUD) during surgery as augmented reality simulations. Thus, the lesion contour and all imaging aspects of the lesion or eloquent anatomical image structures could simultaneously be correlated with tissue structures and manipulated with surgical instruments guided by the augmented reality, Fig. 3. Patient positioning, patient transfer between the operating room and the MRI scanner room as well as intra-operative image modalities were described in detail in a previous publication (Roessler et al., 2024).

### 2.6 Resection strategy

The microsurgical resection itself was carried out according to the borders of the structural abnormalities in the MRI scans. Hypometabolic areas of the PET were also resected if they were larger than the structural abnormalities and an eloquent area was not at risk to be damaged. Resection volumes after sEEG were manually segmented due to the positive contacts of the depth electrodes and with frontal or temporal pole included in the resection, if it was close to those areas.

### 2.7 Retrospective study design and possible bias

The patient cohort investigated were consecutive pediatric neurosurgical patients within a 3 years study period (10/2020-10/2023) and not selected for navigated resection applying augmented reality enhanced functional neuronavigation. The main goal of this study was to perform a feasibility study, to demonstrate the applicability of augmented reality for pediatric epilepsy surgery. Of course, a bias cannot be ruled out concerning the Center indications for resective surgery according to an inhouse multidisciplinary long experienced epilepsy surgery team and indications.

## 3 Results

There were no adverse events while using AR enhanced neuronavigation. Moreover, the study revealed practicle advantages of including AR into the microsurgical part of the surgeries. First, on-line targeting of the lesions and visualization of eloquent brain areas next to the lesion allowed a straight forward surgical entry to the lesions. Second, by using augmented reality data within the microscope, on-line recognition of eloquent tissue was provided during the dissection of the lesions via fMRI/DTI inclusion to avoid injury of eloquent cortex and tracts. Additionally, contour-guided resection of lesions even in cases with unclear visual delineation like in sEEG spatial information of a putative epileptogenic zone, was provided. Moreover, by using intraoperative MRI scans, the new scans and segmentation of resection cavities and consequent augmentation of the second look surgery by implementing the intraoperative imaging data as AR allowed the estimation of the resection amount at the end of surgery, correcting the brain shift.

In 36 patients (83.7%) a total resection of the lesion was achieved. In 38 of the 43 patients (88.4%), an intraoperative MRI was performed, which resulted in 3 patients (7.9%) in a second look surgery, where residual lesions were resected. Altogether, 56% of patients had a follow up of more than one year and most of them had still ASM. Out of them, 24 patients (83.3%) displayed a favorable seizure outcome with ILAE 1 (with 75% beeing completely seizure free since the surgery- ILAE 1a). From the 4 patients not seizure free, 2 had incomplete resections of a FCD2b and are investigated for re-surgery and 2 had a mMCD (mild malformation of cortical development), estimated as diffuse lesions according to epileptological reinvestigation.

Concerning complications, 6 patients experienced a transient hemiparesis due to the vicinity of the lesion to motorcortex or internal capsule, which was respected but irritated due to the close manipulation to these structures. Accordingly, this immediate motor deficit resolved within the next 3 months (14%). Two patients experienced a permanent upper quadrantanopia at the contralateral side to the surgery, due to lesioning of parts of Mayers loop because of lesion extension into the dorsal temporal lobe (4.7%). One 2 year old child needed a subdural-peritoneal shunt because of progredient subdural hygroma (2%), which is seldomly seen in very young children due to hemorrhagic CSF postoperatively. No acute complications like postoperative hemorrhages leading to surgical revision were encountered.

## 4 Discussion

Augmented reality (AR) real time surgical neuronavigation proofed usability and efficacy in this retrospective study in microsurgery of 43 children with drug resistant epilepsy (DRE). AR facilitated targeting and outlining epilepsy lesions during resection as well as intraoperative real-time MR image guidance using navigated surgical instruments. Altogether, 83.3% of patients with a FU of more than one year operated for drug resistant seizures achieved a favorable seizure outcome ILAE Class1 (75% ILAE Class1a), comparable to other reports for resection of dysplastic lesions or LEATs (Mann et al., 2022; Pelliccia et al., 2017; Racz et al., 2021; Ristic et al., 2020; Roessler et al., 2016; Shawarba et al., 2024).

Transferring image information on to the surgical field for online-neuronavigational guidance using AR is a new development adapted from neurosurgical training and surgical interventions in other fields of surgery. Therefore, sparse literature about clinical application in epilepsy surgery is available (Zhou et al., 2024). Recent comprehensive reviews best highlightens the evolution and the state of the art of AR in neurosurgery (Cannizzaro et al., 2022; Mishra et al., 2022). Moreover, new generation operating microscopes can be equipped with navigation tools and polychromatic displays for implementing multimodal image information and 3-plane MRI images (Fig. 1) (Grote et al., 2024). Targeting the lesions with contour guidance via the polychromatic microscope display was already successfully used by the authors who have pioneered this field in the past (Roessler et al., 1997, 1998c). This allows on-line targeting of the epileptic lesions and epileptic foci defined by images and electrophysiological measurements by depth electrodes in the investigated drug-resistant pediatric epilepsy patients in this study (Fig. 2). This was also recently beneficially applied and reported by Grote et al. for extratemporal surgery, but only in 10 adult patients (Grote et al., 2024). These authors used contour guided resections, but without simultaneous 3- plane MRI guidance. In contrast, augmenting the resection by virtual reality images within the microscope is a new development now successfully and routinely applied during pediatric epilepsy surgery during the last 3 years at our institution (Fig. 2).

Not only on-line targeting of lesions seem to be safely available by using AR, but also sparing eloquent brain areas outlined in the neuronavigational images displayed virtually within the eyepiece of the microscope may lead to avoid complications. The complication rate was low in this series compared to the literature (0% permanent motor or speech deficits, 14% temporary hemiparesis, 7% upper quadrantanopia and 2% shunt implantation) (Behrens et al., 1997), contrasting with a seizure outcome which was reasonable good (Englot et al., 2013; Guo et al., 2024), especially when considering that 2/3 of the patients had extratemporal pathologies and ¼ were MRI negative epileptic zones. In contrast, 15% surgical complications and 5% permanent neurological complications and up to 50% quadrantanopia are normally expected in epilepsy surgery according to experiences in large series of operated patients (Behrens et al., 1997).

Additionally, this AR conducted surgery allowed resection of affected tissue without obvious haptic differentiation and estimation of the resection amount at the end of surgery. All different advantages are not new for resection strategies using neuronavigation systems and image-guided surgery (Roessler et al., 2016), but the implementation of augmented reality by using the 3-plane MRI images as direct feedback during resection is a new approach and has not been described for pediatric epilepsy surgery till now.

## 5 Study limitations

The retrospective study design and the absence of a control group operated without the AR application are the main limitations of the study. According to the modernization of our OR by implementing a two-room intraoperative MRI Suite and modern surgical microscopes and navigation systems in 2020, no parallel patient group with comparable demographic data is available. Additionally, the effect of AR application is not quantitatively measurable and an intuitive approach, additionally, the good seizure outcome reported here is very common in dysplastic lesions and epilepsy associated tumors (Mann et al., 2022; Pelliccia et al., 2017; Racz et al., 2021; Ristic et al., 2020; Roessler et al., 2016; Shawarba et al., 2024). Therefore, results of this study have to be considerate very cautiously, especially the seizure outcome, which is a highly multifactorial result of the careful and extensive preoperative investigation of the patient as well as of the application of an appropriate surgical strategy and the cautious microsurgical technique, allowing hardly reliable conclusions on a single surgical application tool used. Additionally, brain shift during resection made the AR overlays inaccurate within the course of resection, which we tried to correct by using intraoperative MRI scans in most of the patients if residual lesions were suspected.

## 6 Conclusions

Nevertheless, in the authors opinion AR supported image guided navigated microscope resection facilitated targeting and removal of lesional as well as non-lesional (sEEG defined) epileptogenic zones in a pediatric epilepsy surgery cohort with low morbidity and good seizure outcome. Moreover, AR navigation supported resection should also be included in the resident training program to introduce residents to be able to process complex AR overlays during microsurgical resection using modern microscopes.

## Author contributions

“Conceptualization, R.K.; methodology, R.K., J.S.; validation, R.K., J.S., M.T., F.M.; formal analysis, F.W.; investigation, X.X.; resources, K.R., M.F.; data curation, M.F.; writing—original draft preparation, J.S., K.R.; writing—review and editing, K.R., M.F.; visualization, K.R., J.S.; supervision, K.R. and C.D., All authors have read and agreed to the published version of the manuscript.

## Institutional Review Board statement

The study was conducted in accordance with the Declaration of Helsinki, and approved by the Institutional Review Board (or Ethics Committee) of Medical University of Vienna (EK Nr: 1542/2024, ARPES Study).

## Funding

“This research received no external funding”

## Declaration of competing interests

There are no conflict of interests of the authors concerning this manuscript. The authors declare that they have no known competing financial interests or personal relationships that could have appeared to influence the work reported in this paper.

## Figures and Tables

**Table:** 

AR	Augmented reality
ASM	Anti seizure medication
DTI	Diffusion tensor imaging
DIG/DIA	Desmoplastic infantil ganglioglioma/astrocytoma
DNT	Dysembryioplastic neuroepithelioma
EZ	Epileptogenic zone
FCD	Focal cortical dysplasia
fMRI	Functional magnetic resonance tomography
GG	Ganglioglioma
HUD	Head up display
HS	Hippocampal sclerosis
IGS	Image guided surgery
ILAE	International league against epilepsy
iopMRI	Intraoperative MRI
LEAT	Long term epilepsy associated tumor
mMCD	Mild malformation of cortical development
MOGE	Mild malformation of cortical development with oligodendroglial hyperplasia
MRI	Magnetic resonance tomography
PET	Positron emission tomography
sEEG	Stereo encephalography
SPECT	Single photon emission computer tomography
SEGA	Subependymal giant astrocytoma
